# Loose Fit Monobloc Versus Cemented Bipolar Radial Head Prosthesis in the Treatment of Radial Head Fractures: A Comparative Study

**DOI:** 10.3390/jcm15155989

**Published:** 2026-08-01

**Authors:** Guglielmo Miele, Alessandro El Motassime, Francesco Farine, Cesare Stefanelli, Guido Bocchino, Omar El Ezzo, Camillo Fulchignoni, Felice Minutillo, Calogero Graci, Giulio Maccauro

**Affiliations:** 1 Department of Orthopaedics, Fondazione Policlinico Universitario Agostino Gemelli IRCCS, 00168 Roma, Italy; guglielmo.miele01@icatt.it (G.M.); francesco.farine01@icatt.it (F.F.); cesare.stefanelli01@icatt.it (C.S.); guido.bocchino01@icatt.it (G.B.); omar.elezzo@policlinicogemelli.it (O.E.E.); camillo.fulchignoni@gmail.com (C.F.); felice.minutillo@policlinicogemelli.it (F.M.); calogerograci@hotmail.it (C.G.); 2 Orthopedics and Traumatology, Università Cattolica del Sacro Cuore, 00168 Roma, Italy

**Keywords:** elbow fracture, radial head fracture, radial head arthroplasty, radial head prosthesis, loose-fit prosthesis, cemented prosthesis

## Abstract

**Background:** Radial head arthroplasty is commonly used for unreconstructable radial head fractures, particularly in cases associated with elbow instability. Both loose-fit monobloc and cemented bipolar radial head prostheses are available, but comparative evidence regarding their clinical and radiographic outcomes remains limited. This study aimed to compare mid-term outcomes between these two implant designs. **Methods:** A retrospective observational study was conducted on 26 patients who underwent radial head arthroplasty for acute radial head fractures between November 2020 and October 2023. Fourteen patients received a loose-fit monobloc radial head prosthesis, while twelve received a cemented bipolar prosthesis. Clinical, functional, and radiographic outcomes were assessed at a minimum follow-up of 24 months. Outcome measures included range of motion, pain, elbow stability, satisfaction, ADL (activities of daily living), IADL (instrumental activities of daily living), SF-12 (12-Item Short Form Health Survey), Oxford Elbow Score, Mayo Elbow Performance Score, DASH (disability of the arm, shoulder and hand score) score, implant loosening, heterotopic ossification, and complications. **Results:** No statistically significant differences were observed between the two groups in pain, satisfaction, range of motion, elbow stability, functional scores, radiographic loosening, or complication rates. The cemented bipolar group showed slightly better flexion–extension and pronation–supination values, whereas the loose-fit group showed a numerically lower rate of elbow instability; however, these differences were not statistically significant. No cases of aseptic loosening, periprosthetic osteolysis, or undesired implant loosening were observed. The complication rate was 36% in the loose-fit group and 33% in the cemented group. One patient in the loose-fit group required revision surgery due to implant overstuffing. **Conclusions:** Both loose-fit monobloc and cemented bipolar radial head prostheses provided acceptable mid-term functional scores; however, the relatively high overall complication rate (33–36%) highlights the need for careful implant selection, particularly with loose-fit implants, to avoid overstuffing and postoperative stiffness. Given the small cohort, these findings do not imply therapeutic equivalence.

## 1. Introduction

Radial head fractures (RHF) are the most frequent fractures of the elbow in the adult population. This fracture type accounts for approximately one-third of all elbow fractures among adults [1].

Nondisplaced fractures of the radial head usually occur in isolation, while displaced fractures are usually associated with ligament tears of the medial collateral ligament (MCL), lateral collateral ligament (LCL), and interosseous ligament. In high-energy injuries, RHFs may be associated with the dislocation of the elbow and forearm. Also, coronoid fractures are often seen with RHFs; this association can affect elbow stability [2]. Isolated RHFs do not cause elbow instability since the radial head is not a main elbow stabilizer; the MCL, LCL, and coronoid process are the main elbow stabilizers, so RHFs affect elbow stability only in case of concomitant lesions of these structures [3].

### 1.1. Radial Head Fractures Classifications

**Figure 1:**
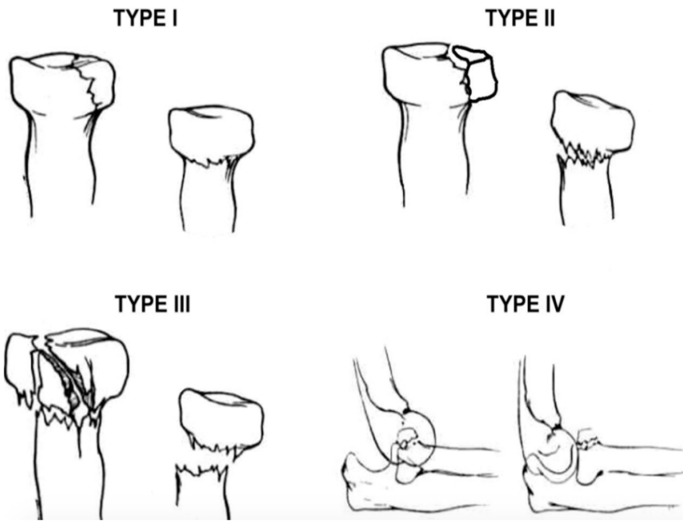
Mason classification modified by Hotchkiss.

In 1954, Mason proposed a RHFs classification that is still widely used; it consists of 3 types of fractures: Type I: nondisplaced or minimally displaced fractures; Type II: fractures with a displacement > 2 mm or affecting more than 30% of the articular surface; Type III: comminuted fractures involving the whole head of the radius [4]. Hotchkiss modified the Mason classification by adding a type IV fracture that consists of RHFs associated with elbow displacement (Figure 1) [5]. Nondisplaced Mason type I fractures or minimally displaced fractures usually do not result in a loss of the elbow range of motion (ROM) and they are treated nonoperatively. Mason Type II fractures can be treated in different ways: non-operative treatment, fragment excision, open reduction and internal fixation (ORIF), radial head excision or radial head arthroplasty (RHA) [2]. Although surgical indications are not unequivocal, nonoperative treatment can be considered for Mason type II fractures if the elbow is stable and there are no constraints on elbow motion. Otherwise, ORIF should be reserved for minimally comminuted fractures with 3 or fewer articular fragments. Fragment excision is an alternative option for Mason type II fractures when a displaced fragment mechanically blocks motion but cannot be internally fixed due to severe comminution or poor local bone quality. Fragment excision may increase the risk of subsequent radial head subluxation, though [1]. If the degree of comminution precludes ORIF, radial head excision is an option for isolated Mason type II fractures in stable elbows; otherwise, if these fractures are associated with MCL or LCL tear or coronoid and/or capitellum fractures, resulting in an unstable elbow, RHA is indicated [2].

Mason type III fractures are non-reconstructable by definition, and so the surgical options include head excision with or without RHA. Though radial head excision alone can improve pain and guarantee a good range of motion, it can also lead to poor forearm rotation, radial shortening with consequent wrist pain, decreased grip strength and early ulnotrochlear arthrosis with elbow pain. Older patients with low functional demands and no ligamentous instability are usually eligible for radial head excision. Otherwise, younger patients with higher functional demand and/or patients with elbow instability should be treated with RHA [1].

### 1.2. Radial Head Arthroplasty

In recent years, RHA has been increasingly accepted as a treatment for patients with RHFs that cannot be managed with ORIF, especially when the RHF is associated with ligamentous lesions causing elbow instability. Radial head replacement’s goals can be divided into short-term and long-term: during the first 2–3 weeks after surgery, the radial head prosthesis (RHP) acts as a spacer, stabilizing the elbow and allowing soft tissues, such as the MCL, LCL, and interosseous membrane, to heal properly. Radial head replacement also allows early elbow mobilization, reducing the risk of stiffness. In the medium and long terms, RHPs contribute to elbow load absorption, protecting the ulno-humeral joint from mechanical overload and avoiding excessive MCL tension during valgus stress. Thanks to these features, RHPs prevent ulnohumeral arthrosis, valgus deformity, and tardive ulnar nerve symptoms [6]. RHA also impedes radial shortening, preventing late-onset wrist pain. RHPs can be classified based on two main structural characteristics: the implant’s head and the stem fixation method. Regarding the head design, RHPs can be classified into monobloc prostheses, which feature a rigid, fixed cup attached directly to the stem, and bipolar prostheses, which incorporate a mobile, articulating cup [7].

### 1.3. Rigid-Fit vs. Loose-Fit Radial Head Prosthesis

Regarding stem fixation within the radial medullary canal, implants are broadly categorized into rigid-fit and intentionally loose-fit systems. Rigid fixation can be achieved with both press-fit stems and cemented stems. Press-fit or cemented stem implants have been developed to replicate the radial head anatomy as closely as possible, providing primary stability and reproducing the physiological radio-capitellar biomechanics and kinematics. However, rigid fixation RHPs are associated with specific mechanical disadvantages: press-fit configurations are associated with a higher complication rate due to symptomatic implant loosening and metaphyseal stress shielding, while cemented-stem implants may be technically challenging to remove if revision surgery is needed [6,8]. Conversely, intentionally loose-fit implants have a circular, smooth stem; smooth-stemmed implants are intentionally designed to be undersized so that the stem has a certain degree of translation and movement within the radial canal. This structural characteristic provides a self-centering mechanism, meaning that during elbow pronation–supination and flexion–extension, the movements between the body canal and loose-fit stem help better accommodate the inevitable minor incongruences between the prosthesis and the lesser sigmoid notch. With this study, we aim to compare the clinical and functional outcomes between loose-fit monobloc RHPs and cemented bipolar RHPs in patients undergoing radial head arthroplasty.

## 2. Materials and Methods

A retrospective observational study was conducted in accordance with the PROCESS guidelines. The study protocol was approved by the Institutional Review Board of the Orthopedic and Traumatology Institute and was performed in compliance with national ethical standards and the Declaration of Helsinki.

Between November 2020 and October 2023, 26 patients underwent surgical treatment with RHA at the Policlinico Agostino Gemelli, Rome. All patients were treated with either a monoblock loose-fit RHP or a bipolar cemented RHP. The choice of prosthesis (loose-fit vs. cemented bipolar) was based on intraoperative assessment and surgeon preference, considering factors such as bone quality and elbow stability. No standardized allocation protocol was used. This represents a potential source of selection bias.

Written informed consent for both surgical procedure and collection of clinical data for research purposes was obtained from all participants at hospital admission and prior to surgery, in accordance with institutional protocols. Inclusion criteria comprised prosthetic replacement following acute RHFs treated with RHA, with a minimum follow-up of 24 months. Exclusion criteria included radial head arthroplasty performed in patients with elbow osteoarthritis, cases in which RHA was performed after failed prior ORIF treatment of RHFs, and a follow-up period of less than 24 months. Using the institutional database, patients meeting the inclusion and exclusion criteria were identified. A total of 26 patients were ultimately included in the study: 14 patients in the loose-fit RHP group and 12 patients in the cemented bipolar RHP group.

The RHFs were assessed using the standard antero-posterior (AP) and latero-lateral (LL) radiographs. All the fractures were classified using the Mason Classification modified by Hotchkiss [5].

### 2.1. Implant Selection

Implant selection was non-randomized and based on a comprehensive intraoperative assessment of bone quality and associated ligamentous injuries. Cemented bipolar components were preferred in patients presenting with severe metaphyseal comminution, partially involving the radial neck, and osteoporotic bone. Conversely, intentionally loose-fitting monobloc implants were selected when the radial neck and metaphyseal bone stock were well preserved. Surgeon preference and system familiarity also guided the choice, introducing an acknowledged selection bias.

### 2.2. Surgical Technique

**Figure 2:**
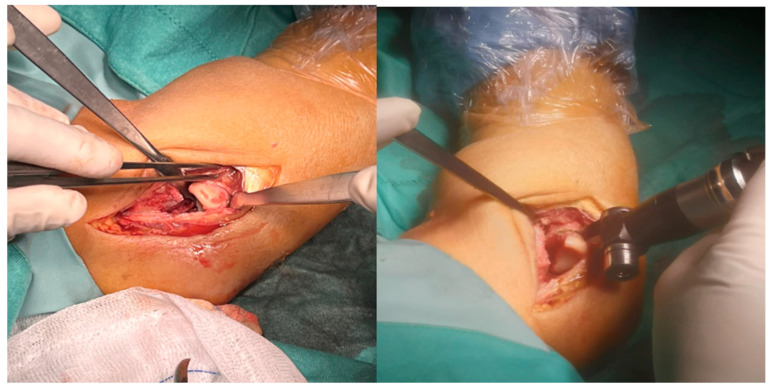
Radial head resection.

**Figure 3:**
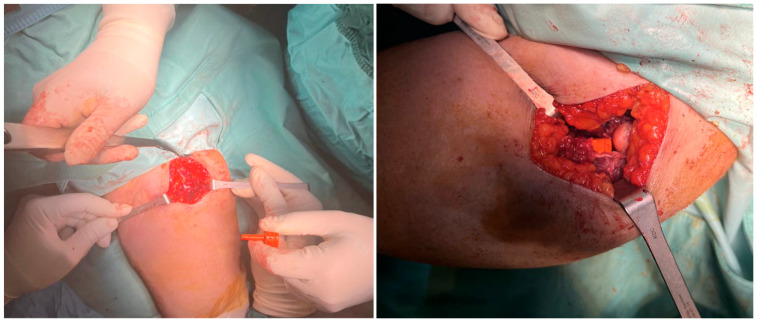
Radial head prosthesis trial and its evaluation.

All procedures were performed by the same surgeon with over 15 years of experience in radial head arthroplasty, using both loose-fit and cemented prosthetic designs, under brachial plexus anesthesia with the patient in a supine position and the affected arm abducted on a suitable support. Preoperatively, antibiotic prophylaxis was administered using Cephazoline 2 g i.v. when not contraindicated. A tourniquet was positioned above the affected elbow joint. A skin incision was performed starting over the lateral supracondylar ridge, 5 cm proximal to the elbow joint. The deep fascia was incised in line with the skin incision. The interval between the triceps muscle posteriorly and the brachioradialis and extensor carpi radialis longus is developed, exposing the lateral humeral epicondyle anteriorly. The dissection is continued through the Kocher interval, passing between the extensor carpi ulnaris and the anconeus muscles. The ligamentous structures are explored and tested to better identify ligamentous lesions. Radial head resection is first performed and all the fragments are removed, making sure no fragment remains in the joint (Figure 2). After radial head resection, radial head height and width are measured to choose the right prosthesis size. When a loose-fit radial head prosthesis is implanted, a depth gauge is then used to probe the depth and the diameter of the radial canal. Once the radial canal has been probed, a broach is used to prepare the radial canal to receive the loose-fit stem. A radiolucent prosthetic trial is then implanted to test stability and range of motion (Figure 3), after which it is radiographically evaluated. If the trial gives satisfactory results, the definitive prosthesis is implanted. The definitive implant is evaluated both radiologically and clinically: varus-valgus stress is applied to the elbow flexed at 20°, ROM is assessed by passive flexion–extension of the elbow and pronation–supination. If the results are satisfactory, then surgical irrigation is performed followed by soft tissue closure.

When a cemented radial head prosthesis was implanted using the appropriate instrumentation, an osteotomy of the radial head was first performed. The medullary canal was then prepared to accommodate the stem. Following this step, the trial radial stem and radial cup were inserted. The height of the coronoid and the alignment of the ulnohumeral joint were considered the references to assess the correct sizing of the trial components. Trial prosthetic components were tested, selecting the appropriate size based on the dimensions of the resected fractured radial head. If dynamic testing confirmed satisfactory results, the radial canal was prepared for the implantation of the definitive cemented components. After a thorough irrigation of the radial medullary canal, a cement plug was placed and low-viscosity polymethylmethacrylate cement was introduced into the canal in a retrograde fashion. The prosthetic stem was then inserted in correct alignment and maintained in position until complete cement polymerization. During the procedure, ligamentous structures are explored and tested to better identify and, if necessary, treat any ligamentous lesions.

**Figure 4:**
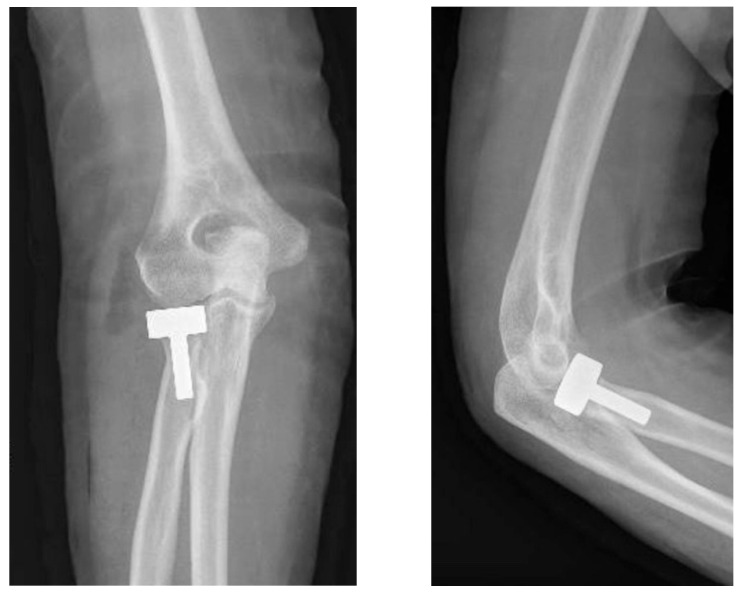
Radiographical AP and LL view of the implanted loose-fit monobloc radial head prosthesis.

**Figure 5:**
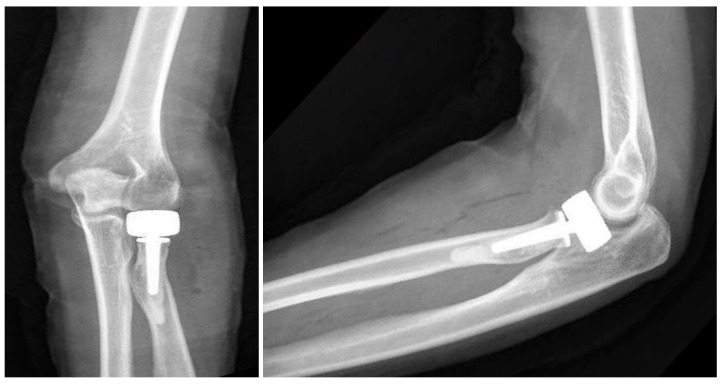
Radiographical AP and LL view of the implanted cemented bipolar radial head prosthesis.

Postoperative anteroposterior and lateral radiographic evaluation of the implant was finally performed (Figure 4 and Figure 5).

### 2.3. Post-Operative Management and Follow-Up

Postoperative management and physiotherapy protocol were the same for both the cemented and loose-fit RHP groups.

For the first two postoperative weeks, patients received celecoxib 200 mg twice daily to prevent heterotopic ossification [9,10].

The first clinical evaluation of patients was carried out approximately 15 days after surgery. Subsequent outpatient clinical and radiographic evaluations were performed at 1, 3, 6, 12, and 24 months. In the postoperative period, all patients were managed with an articulated elbow brace positioned at 90° of flexion, allowing a controlled range of motion of 30° in both flexion and extension. Patients were instructed to remove the brace three times daily, starting on postoperative day 1, to perform pronation–supination exercises as tolerated based on pain levels. At 20 days after the index procedure, patients were advised to gradually discontinue use of the elbow brace and initiate unrestricted elbow mobilization.

### 2.4. Physiotherapy Protocol

In cases where patients exhibited apprehension about initiating free elbow mobilization, they were encouraged to work with a physical therapist to progressively regain full range of motion (ROM). Approximately one month after surgery, patients were instructed to work with their physical therapists to begin upper-limb loading exercises aimed at strengthening the upper-extremity musculature.

The clinical assessment included a record of the patient’s level of pain using a Visual Analogue Scale (VAS). We also evaluated the elbow range of flexion–extension and pronation–supination, the elbow stability and strength.

Standard AP and lateral elbow radiographs were evaluated for congruity of the radial head with the capitellum, capitellar osteopenia, heterotopic ossification, size of prosthesis and periprosthetic loosening. To evaluate the size of the implanted prosthesis, we compared the width of the medial and lateral ulnohumeral joint spaces of each treated elbow with the contralateral elbow. If the width of the lateral ulnohumeral joint was increased compared with the contralateral elbow, the prosthesis was considered oversized. The scientific literature has proved that normal elbow function can be achieved with a flexion–extension ROM of 100° and a pronation–supination arc of 50°, so we consider elbow stiffness the condition where patients can not achieve these arcs of motion [11].

### 2.5. Clinical Assessment of the Elbow

The primary outcome measures defined for this study were the elbow ROM, evaluating individual flexion–extension and pronation–supination arcs, the Mayo Elbow Performance Score (MEPS), and the VAS for post-operative pain assessment. Secondary outcome measures included clinical elbow stability, residual strength deficits, and a battery of functional and quality-of-life questionnaires consisting of the Disabilities of the Arm, Shoulder and Hand (DASH) score, the Oxford Elbow Score (OES), the 12-Item Short Form Health Survey (SF-12), the Activities of Daily Living (ADL) score, and the Instrumental Activities of Daily Living (IADL) score [12,13,14,15,16,17].

At each follow-up visit, elbow function was systematically assessed. Range of motion was evaluated by measuring flexion–extension and pronation–supination ROM. Elbow stability was clinically assessed using varus and valgus stress tests performed with the elbow flexed at approximately 20–30 degrees. Finally, we asked patients to report strength deficits in both flexion–extension and pronation–supination of the affected elbow compared with the contralateral elbow; patients were asked to describe the loss of strength as severe, moderate, mild, or no loss of strength based on their ability to lift a 2 kg weight.

Regarding the secondary functional questionnaires, the ADL, IADL, and SF-12 scores were calculated to assess and compare patients’ overall condition both prior to the injury and post-operatively. The remaining elbow-specific functional parameters—including the OES and the DASH score—were systematically recorded during the clinical follow-up tracking to evaluate targeted joint recovery and residual disability.

### 2.6. Complications

Complications were defined as any adverse deviation from the normal postoperative course and classified as minor (transient neuropraxia, mild pain) or major (persistent stiffness, secondary instability, or any condition requiring revision surgery).

### 2.7. Statistical Analysis

Data were collected using the Excel programme (Microsoft, Redmond, WA, USA). Due to the limited sample size, no a priori power analysis was performed. Continuous variables were tested for normality using the Shapiro–Wilk test. Given the non-normal distribution, comparisons between groups were performed using the Mann–Whitney U test. Categorical variables were analyzed using Fisher’s exact test. Statistical significance was set for *p* < 0.05.

## 3. Results

The study retrospectively evaluated 26 patients undergoing RHA; 14 patients were included in the loose-fit monobloc RHP group, and 12 patients were included in the cemented bipolar RHP group.

In the loose-fit RHP group, 5 patients were men and 9 women, with a mean age of 54.8 ± 14.6 years, and were followed for an average of 27.6 months (±6.7).

The cemented RHP group included 5 male and 7 female patients, whose overall mean age was 56.8 ± 4.8 years; they were clinically followed for an average of 27.5 months.

Patients’ demographic data are reported in Table 1.

Of the 26 patients included in this study, 19 had a Mason type III fracture (10 cases in the loose-fit RHP group and 9 in the cemented RHP group) and 7 had a Mason type IV fracture (4 cases in the loose-fit RHP group and 3 in the cemented RHP group). Concomitant injuries were frequent: in the Mason type IV subgroups, 4 patients in the loose-fit group and 3 in the cemented group presented with acute elbow dislocation associated with lateral ulnar collateral ligament (LCL) tears; 1 patient in the cemented group reported a ‘terrible triad’ injury pattern with an associated coronoid fracture requiring fixation.

No statistically significant differences were found between the loose-fit and cemented groups in any of the primary or secondary outcomes, except for SF-12 PCS.

At the final follow-up, patient satisfaction was evaluated using a VAS ranging from 0 to 10; the overall average satisfaction was 8.02 (±2.74). The loose-fit RHP group reported a satisfaction rate of 8.1 (±1.9), while the cemented RHP group reported a satisfaction rate of 7.93 (±3.36). The overall average VAS was 2.2 (±2.58). The loose-fit RHP group presented with a VAS score of 2.33 (±2.11), while the cemented RHP group presented with a VAS score of 2.04 (±1.88). During the last follow-up visit, we evaluated each patient’s elbow range of motion; the patients’ elbow mobility has been reported for both the loose-fit RHP and cemented RHP groups in Table 2.

Another parameter we monitored during the follow-up visits was elbow flexion and extension strength, and we found that in the loose-fit RHP group, 4 patients reported mild strength deficit at flexion–extension, 1 patient presented with a moderate strength deficit at flexion–extension and 1 patient presented with a severe strength deficit at flexion–extension. In the cemented RHP group, 2 patients exhibited moderate strength deficit, while 1 patient presented with severe strength deficit at flexion–extension. We also evaluated pronation–supination strength: in the loose-fit RHP group, 3 patients presented with mild strength deficit at pronation–supination, 1 patient exhibited moderate strength deficit at pronation–supination, and 1 patient exhibited severe strength deficit at pronation–supination. On the other hand, in the cemented RHP group, 2 patients presented with mild strength deficit and 2 patients presented with moderate strength deficit at pronation–supination.

Radiographically, there was no evidence of implant loosening or undesired loosening in both loose-fit and cemented RHP groups. A certain degree of incongruence between the implant and radial canal was considered normal in the loose-fit RHA group; the gap between the loose-fit prosthesis and the radial canal was always inferior to 2 mm. We also looked for any heterotopic calcifications, and we found that 2 patients presented with heterotopic calcification in the loose-fit group, with a rate of 14%. On the other hand, 1 heterotopic ossification was found in the cemented RHP group. In both the cemented and loose-fit RHP groups, there was no radiographic evidence of periprosthetic osteolysis.

Patients included in our study reported overall good functional scores: functional scores of both loose-fit and cemented RHP groups have been reported in Table 3.

In the loose-fit RHP group, the complication rate was 36%: 2 patients presented with elbow stiffness at the latest follow-up, 1 patient presented with persistent wrist pain after surgery, and 2 patients exhibited persistent numbness of the forearm. One of the 2 patients with elbow stiffness underwent revision surgery because the prosthesis was overstuffed, so the revision rate in the loose-fit group was 7%.

In the cemented RHP group, the complication rate was 33%: 2 patients reported radial nerve deficit after surgery and 1 patient exhibited hand numbness and motor deficit after the procedure, with a full recovery of both sensitive and motor function after 4 months from surgery. Another patient also reported numbness in the back of the hand after surgery. The symptoms resolved 3 weeks after surgery. No patient in the cemented RHP group required revision surgery.

## 4. Discussion

The current study retrospectively evaluated 26 patients who underwent RHA using monobloc loose-fit RHPs and bipolar cemented RHPs for the treatment of acute RHFs, reporting the midterm clinical and radiographic outcomes. Our analysis was mostly focused on the impact that such surgery has on the patient’s functional activities of daily living and whether loose-fit and cemented RHPs yield any difference in terms of clinical outcomes. We also aim to compare our results to the data provided by scientific literature about rigid-fit and loose-fit RHPs. Overall, the outcomes can be considered positive, with no statistically significant differences detected between the two study groups. However, our small sample size limits the statistical power to definitively demonstrate therapeutic equivalence between loose-fit monobloc and cemented bipolar radial head prostheses with respect to clinical, functional, and radiographic outcomes at mid-term follow-up. Despite this limitation, our data indicate that patients in both groups generally achieve high satisfaction levels and experience minimal restrictions in daily life, with some even returning to their sports activities without limitations. Even patients with a lower MEPS, DASH score, or OES still reported high scores on the ADL and IADL questionnaires; these data suggest that a reduction in the affected elbow functionality does not necessarily translate into a reduction in the capacity to accomplish daily tasks. On the other hand, lower elbow functional scores are associated with lower SF-12 MCS scores since a deficit in elbow functionality may affect the way patients perceive their physical and mental health. In our study, female patients in both the loose-fit RHP group and the cemented RHP group showed SF-12 MCS values below the general population mean; however, no statistically significant difference was observed between the two groups.

Le Mapihan M et al. in a retrospective study on 56 patients with RHFs treated with rigid-fit cemented RHPs reported a mean MEPS of 84.1 and a mean DASH score of 18.2 [18]. Yang G. et al. in their systematic review compared elbow functional score in RHFs treated with cemented stem RHPs and uncemented stem RHPs: patients treated with cemented RHPs reported a mean MEPS of 82.5, a mean DASH score of 20.25, and a mean VAS of 1.7. On the other hand, patients treated with uncemented RHPs reported a mean MEPS of 85, a mean DASH score of 18.6 and a mean VAS of 1.75 [19]. So, according to scientific literature, there is no significant difference between cemented RHPs and uncemented RHPs in terms of clinical outcome and residual pain. The data collected during our study are consistent with reports in the scientific literature: although patients treated with cemented prostheses generally demonstrated higher functional scores than those treated with loose-fit prostheses, no statistically significant differences were observed between the two groups. The only outcome that showed a statistically significant difference between the male study groups was the SF-12 PCS score, with patients treated using cemented RHPs reporting significantly higher scores.

Also, the scientific literature does not provide consensus on whether loose-fit and cemented prostheses differ in mobility outcomes. Le Mapihan M et al. in their retrospective study reported the following ranges of motion in patients treated with cemented RHPs: 126° in flexion, 9° of extension, 76° of pronation and 79° of supination [18]. From the data we collected, it emerged that there was no statistically significant difference between the two groups in terms of elbow range of motion, though patients treated with cemented RHPs show a slightly better range of elbow pronation–supination and flexion–extension compared with patients treated with loose-fit implants.

Viveen et al. reported that loose-fit prostheses report a higher incidence of failure due to stiffness when compared with rigid-fit prostheses, and among loose-fit prostheses, monobloc designs failed more often than modular designs [8]. Heijink A. et al., instead, in their meta-analysis, stated that the model of the prosthesis does not significantly affect the flexion–extension and pronation–supination arc [20]. In our case series, only 2 patients exhibited elbow stiffness at the last follow-up visit, both in the loose-fit group. Specifically, the first patient demonstrated a flexion–extension ROM of 45° and almost no pronation–supination. The same patient presented with extensive heterotopic ossification at radiological examination, which may explain the clinical stiffness. Despite the limitation in elbow movement, the patient reported good postoperative VAS, ADL and IADL scores and did not require any further treatment. The other case of elbow stiffness was due to the positioning of an overstuffed loose-fit RHP. Antoni M. et al., in a retrospective study including 58 patients undergoing RHA using bipolar and monopolar rigid-fit prosthesis, reported an instability rate of 20.7% [7]. In our study, the loose-fit RHP group yielded an instability rate of 14%; on the other hand, in the cemented RHP group, there were 3 cases of elbow instability at the last follow-up visit, with an instability rate of 25%. Viveen J et al. in their meta-analysis also reported higher rates of elbow instability after RHP using rigid-fit prosthesis when compared with intentionally loose-fit prosthesis [8].

Loose-fit RHPs act as a spacer to allow proper healing of the surrounding soft tissues around the elbow, a role that complements the self-centering mechanism; during pronation–supination and flexion–extension, the movements between the loose-fit stem and the radial medullary canal help to better center the prosthesis, reducing as much as possible the incongruencies between the prosthesis and the lesser sigmoid notch. Both these mechanisms may reduce the risk of aseptic loosening and elbow instability: Viveen J. et al. also reported that aseptic loosening represents 30% of all failures in RHA surgery: of the 46 reported cases of aseptic loosening, only six occurred in patients treated with a loose-fit prosthesis, whereas the remainder involved individuals who had received a rigid-fit implant [8]. None of the patients included in our study presented with aseptic loosening of the implant. Out of the 26 patients included in our study, only 1 patient belonging to the loose-fit RHP needed revision surgery since the prosthesis was overstuffed. Heijink A. et al., in their meta-analysis, reported no significant difference in revision rates between loose-fit RHPs and rigid-fit RHPs [20]. On the other hand, Kachooei et al. in their study reported different removal/revision rates for different types of prosthesis: cemented stem RHPs yielded a revision rate of 4.0%, uncemented smooth stem RHPs reported a revision rate of 8.2%, while RHPs with rigid-fit stems reported a revision/removal rate of 11.3% [21]. Finally, scientific literature does not provide precise data about complication rates in RHAs. One of the main reasons why it is hard to find data about complication rates is that there is no complete agreement on whether radiographic complications should be included in the complication rate or not. In our study, we did not include radiographic complications such as heterotopic calcifications in the overall complication rate; the complication rate in the loose-fit RHP group is 36% while the complication rate in the cemented RHP group is 33%. The overall complication rate is 31%, which appears slightly higher compared with some reports in the literature. In a systematic review, Heijink A. et al. reported 182 complications over a population of 727 patients who underwent RHA; in their study, Heijink et al. included both rigid-fit prosthesis and loose-fit prosthesis with an overall complication rate of 25% without a statistically significant prevalence of complications in the loose-fit RHP group or cemented RHP group [20]. In our study, even though the loose-fit RHP group reported a higher complication rate than the cemented RHP group, this difference was not statistically significant (*p*-value: 0.5). This high complication rate deserves careful analysis. In the loose-fit group (36%), complications were mainly mechanical; specifically, a case of heterotopic ossification and a case of overstuffing led to post-operative stiffness, which had a measurable negative impact on individual functional outcomes (MEPS and DASH) and, in the case of overstuffing, necessitated revision surgery (7%). Conversely, complications in the cemented group (33%) were primarily neurological, specifically transient radial nerve neuropraxia likely related to surgical traction, which resolved completely within 4 months without impacting long-term function. These findings emphasize that while neurological events may be transient and strictly related to surgical technique, loose-fit designs demand absolute sizing precision in implant selection to prevent alteration of joint kinematics.

This study has several limitations; first, its retrospective design introduces a potential risk of selection bias. Another limitation is represented by the small sample size. Also, the follow-up period does not allow for a proper evaluation of long-term outcomes and complications. Finally, the heterogeneity of the study population and the inclusion of different Mason fracture types represent major confounding factors. Finally, the absence of a standardized protocol for implant selection may have introduced treatment allocation bias. Future investigations with expanded cohorts and longer follow-up, ideally at five years, are warranted to better evaluate long-term outcomes.

## 5. Conclusions

In conclusion, both loose-fit monobloc and cemented bipolar radial head prostheses provided good mid-term functional scores in the treatment of unreconstructable radial head fractures. No statistically significant superiority of one design over the other was demonstrated. However, the relatively high complication rate mandates caution. Meticulous intraoperative sizing remains crucial, particularly for loose-fit designs, to prevent overstuffing and subsequent joint stiffness. Due to the small sample size and retrospective nature of this study, further large-scale randomized trials are required to fully evaluate the long-term outcomes and true equivalence of these implants.

## Figures and Tables

**Table 1:** Demographic data; the reported values are mean values; FU: follow-up; BMI: body mass index; CCI: Charlson comorbidity score; RHP: radial head prostheses.

	Loose-Fit RHP (*n* = 14)	Cemented RHP (*n* = 12)	*p*-Value
Sex (M/F)	5/9	5/7	0.68
Age (years)	54.8 ± 14.6	56.8 ± 4.8	0.72
Follow-up (months)	27.6 ± 6.7	27.5 ± 5.9	0.95
Smokers (*n*)	5	5	0.64
BMI	29.1 ± 3.2	27.7 ± 2.5	0.58
CCI	5.2 ± 1.1	5.5 ± 1.0	0.61

**Table 2:** Clinical outcomes; VAS: visual analogue scale; RHP: radial head prostheses..

Clinical Elbow Parameters	Loose-Fit RHP Mean Value (SD)	Cemented RHP Mean Value (SD)	*p* Value
Pronation	63.93 (±16.50)	71.25 (±10.83)	0.29
Supination	66.43 (±19.23)	73.42 (±10.20)	0.33
Flexion	115.71 (±17.49)	122.75 (±11.30)	0.41
Extension	14.14 (±9.64)	10.00 (±6.03)	0.24
Average VAS	2.33 (±2.11)	2.04 (±1.88)	0.62
Stable elbow	12 stable/2 unstable	9 stable/3 unstable	0.19
Satisfaction rate	8.1 (±1.9)	7.93 (±3.36)	0.77

**Table 3:** Functional outcomes of the 26 patients included in the study reported as mean values; ADL: index of Independence in Activities of Daily Living; IADL: Instrumental Activities of Daily Living Scale; PCS: Physical Score; MCS: Mental score; OES: Oxford Elbow Score; MEPS: Mayo Elbow Performance Score; DASH: Disability of the Arm, Shoulder and Hand score; RHP: radial head prostheses.

Functional Score	Loose-Fit RHP Pre-Op	Loose-Fit RHP Post-Op	Cemented RHP Pre-Op	Cemented RHP Post-Op	Post-Operative *p* Value
ADL	6	6	6	6	-
IADL	8	7.4 (±0.4)	8	7.84 (±0.22)	0.7
SF-12 M	PCS: 57.09 (±0.80); MCS: 56.58 (±0.9.92)	PCS: 47.61 (±1.31); MCS: 48.74 (±2.23)	PCS: 58.93 (±1.54); MCS: 55.42 (±1.44)	PCS: 52.11 (±2.23); MCS: 49.58 (±3.68)	PCS *p* value: 0.008 MCS *p* value: 0.7
SF-12 F	PCS: 53.84 (±2.93); MCS: 45.78 (±1.74)	PCS: 48.15 (±1.67); MCS: 43.21 (±2.34)	PCS: 51.97 (±2.88); MCS: 48.82 (±2.91)	PCS: 49.75 (±3.76); MCS: 43.54 (±3.47)	PCS *p* value: 0.4 MCS *p* value: 0.6
OES	NA	34.17 (±8.3)	NA	35.85 (±9.7)	0.5
MEPS	NA	74.5 (±13.2)	NA	77.11 (±16.9)	0.3
DASH	NA	27.23% (±13.7)	NA	24.55% (±8.28)	0.8

## Data Availability

The datasets used and/or analyzed during the current study are available from the corresponding author on reasonable request.

## Abbreviations

MCL: medial collateral ligament; LCL: lateral collateral ligament; RHF: radial head fracture; ORIF: open reduction and internal fixation; RHA: radial head arthroplasty; RHP: radial head prosthesis; ROM: range of motion; VAS: visual analogue scale; ADL: activities of daily living; IADL: instrumental activities of daily living; OES: Oxford elbow score; MEPS: Mayo elbow performance score; DASH: disability of the arm, shoulder and hand score.
